# Oswaldo Gonçalves Cruz: the character, the scientist, the academician

**DOI:** 10.1590/0037-8682-0313-2020

**Published:** 2020-08-21

**Authors:** Cláudio Tadeu Daniel-Ribeiro, Ana Luce Girão Soares de Lima

**Affiliations:** 1 Instituto Oswaldo Cruz, Laboratório de Pesquisa em Malária, Pavilhão Leônidas Deane, Rio de Janeiro, RJ, Brasil.; 2 Casa de Oswaldo Cruz, Centro de Documentação em História da Saúde, Departamento de Arquivo e Documentação, Rio de Janeiro, RJ, Brasil.

**Keywords:** Oswaldo Gonçalves Cruz, Biography, Brazilian *Academia Nacional of Medicina*, Instituto Oswaldo Cruz, Brazilian Public Health, Yellow fever, Malaria, Smallpox and Plague

## Abstract

The present work analyses some particular aspects of Oswaldo Cruz's unique biography, valuing his work, which was built along a successful physician and scientist professional trajectory and also as a courageous and fortunate formulator of public health policies and of fight strategies against the epidemics that seasonally affected the city of Rio de Janeiro at the beginning of the 20th century. The authors also dwell on his legacy as Head scientist and manager of the Institute that bears his name and became the template for experimental research and medicine in Brazil and the bedrock of the *Fundação Oswaldo Cruz*, one of the most important Brazilian Institutions devoted to teaching, research, development and production in health. This heritage made possible to overcome the existing dissensions between doctors and scientists to build a sanitary movement committed to the major health problems in Brazil. Finally, the paper explores some features of the character and reports some of his moments during his passage, as a Full Academician, at the Brazilian *Academia Nacional de Medicina.*

Oswaldo Gonçalves Cruz’s (1872-1917) Opera Omnia^1^ is 700 pages and his biography by Egydio Sales Guerra (1860-1951)^2^ is almost as long as it. In addition, a statement made by Ezequiel Caetano Dias (1880-1922), five years after Cruz’s death, reassures us: “The Oswaldo Cruz's individuality will hardly find anyone who retraces it in all its exquisite lines”^3^. We will, therefore, not dare to bring you at the brink of exhaustion nor try to minutely describe Oswaldo Cruz’s work, even restricting our scope to his research accomplishments. Writing^a^ about our illustrious fellow researcher, who lived for only 44 years an intense life and achieved such an outstanding and meaningful work, is a practice of learning and reflection, as well as humility and projection. Additionally, it represents an opportunity - especially appropriate in these hard times - to examine the new paths of science and public health.

It is not difficult nowadays to make out between a healthcare practitioner and a scientist. Although, on the other hand, in the case of Oswaldo Cruz, the undertaking of distinguishing the researcher and the public health physician is abstruse. Thus, we praise the talk given by our fellow Paulo Gadelha, past President of Fiocruz, at the Brazilian *Academia Nacional de Medicina (ANM)* ^4^, making clear that such a difficulty is not exclusively ours... “Oswaldo was a man who had a lot of concerns and definitively dedicated himself to them almost all the time”.

Oswaldo Cruz was the son of Bento Gonçalves Cruz (1845-1892), a physician from Rio de Janeiro, and Amália Taborda Bulhões (1851-1922). In 1866, a third-year medical student, Bento joined the Brazilian Army and served for a brief period as a volunteer physician during the Paraguayan War (1864-1870). He was then named 2^nd^ surgeon in Asuncion’s Hospital, where Navy war-wounded received medical care, and was also responsible for the 3^rd^ ward in the same hospital. In appreciation for his service, he was awarded the *Campanha do Paraguai* Medal^5^. After returning home and finishing the medical course, he moved to São Luiz do Piratininga, a small town in São Paulo state, in order to pursue his nest egg. 

The town was then becoming an important farming hub and offered him an opportunity to settle in and set up a number of regular patients. After renting a beautiful house called *Chácara do Dizimeiro* (Figure 1), he returned to Rio and married his cousin Amália. The family lived in São Luiz till 1877. Oswaldo Cruz, the older of six children, was born in 1872. 

**FIGURE 1:**
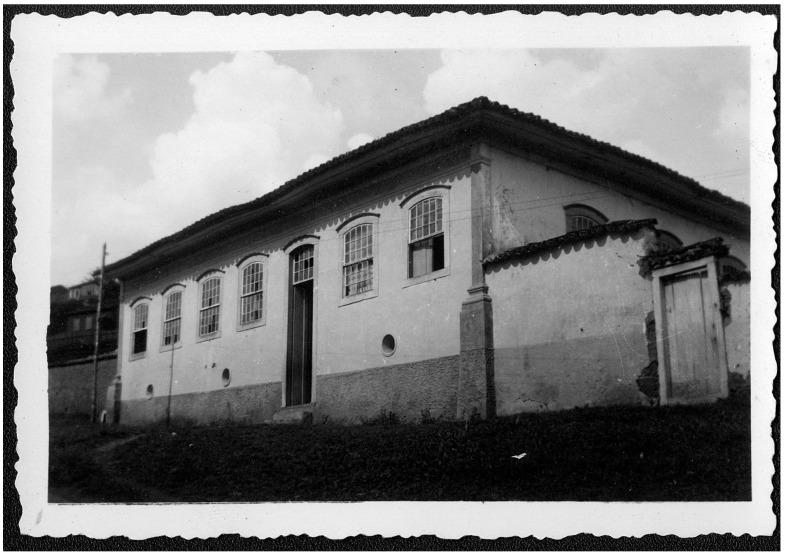
*Chácara do Dizimeiro* is the name given to the house where Oswaldo Cruz was born, in São Luiz do Paraitinga, in the *Vale do Paraíba* (valley of the Paraíba river), São Paulo State, in 1872. He lived there until 1877, when the family moved to a house in *Jardim Botânico*, Rio de Janeiro. The *Chácara* was located at the frontier of the urban area with the rural space where the farms were situated and part of Dr. Bento clientele lived (photo from the *Casa de Oswaldo Cruz* collection).

Back to Rio de Janeiro, the family moved to *Jardim Botânico*, then a remote borough belonging to the parish of Gávea and recently connected to the urban network by means of streetcars. Oswaldo Cruz was at the time a five-year-old boy. Bento settled up a doctor’s office at his own house^b^, while providing medical assistance to the workers of a textile factory (*Fábrica de Tecidos Corcovado*, Figure 2). Bento worked there virtually till his last days, being replaced by his son. Oswaldo thought he couldn’t even miss a single work day in the factory, as he considered he was in charge of his father’s job.

**FIGURE 2:**
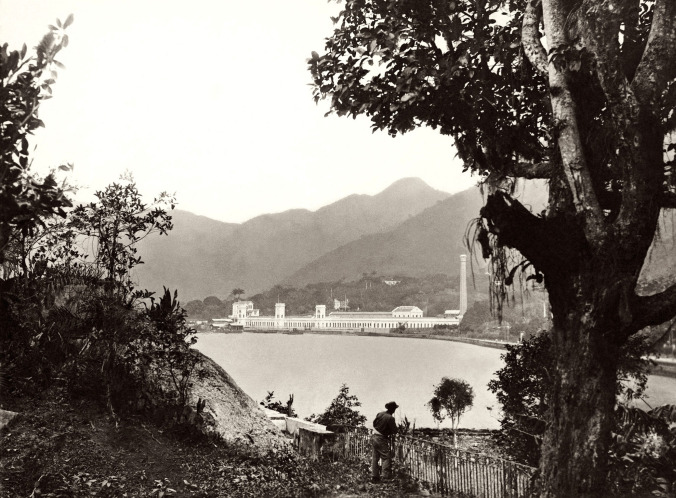
View of the Corcovado fabric factory from the Fonte da Saudade St. The factory was on the Jardim Botânico St., then on the edge of Rodrigo de Freitas Lagoon and ran from the Martins Lage’s mansion (today *Parque Lage*) to Faro St. (photo by Marc Ferrez, *Instituto Moreira Sales* collection).

In 1886, Bento was appointed member of the newly created *Inspetoria Geral de Higiene*, which replaced the *Junta Central de Higiene e Saúde Pública*, after the public health services of the Imperial Court underwent a complex overhaul. The other members of the new inspectorate bureau were the physicians Agostinho José de Souza Lima, Francisco Marques de Araújo Góes and José Ricardo Pires de Almeida; the post of *Inspetor Geral de Higiene* was held by João Batista dos Santos, Baron of *Ibituruna* (1828-1911). The main objective of that department was to promote the basic sanitation of Rio de Janeiro, tackling the yellow fever and smallpox outbreaks that used to plague the city almost every year, and to prevent the arrival of the cholera epidemic in the then Brazilian capital. After the Proclamation of the Republic, in 1889, Dr. Benjamim da Rocha Faria (1853-1936), professor at the School of Medicine of Rio de Janeiro, became the Inspector General. He remained on the job until 1892, when he was replaced by Bento Gonçalves Cruz^6^.

The period while Bento remained in office overlapped with Oswaldo Cruz’s admission to the *Faculdade de Medicina do Rio de Janeiro* (Figure 3), in 1887. During the course, the young man gradually got interested in experimental medicine and microbiology. He benefitted as well from the changes in the Faculty of Medicine required by the so-called “Saboia Reform” of 1884^7^, which made the practical teaching of the subjects mandatory - several laboratories were created in the institution. During the second year of medical school, Oswaldo Cruz was named auxiliary lab preparator in the Hygiene Laboratory, headed at the time by Benjamim Rocha Faria. Two years later, when the laboratory was made into the National Institute of Hygiene, Oswaldo Cruz was promoted to the grade of assistant.

**FIGURE 3:**
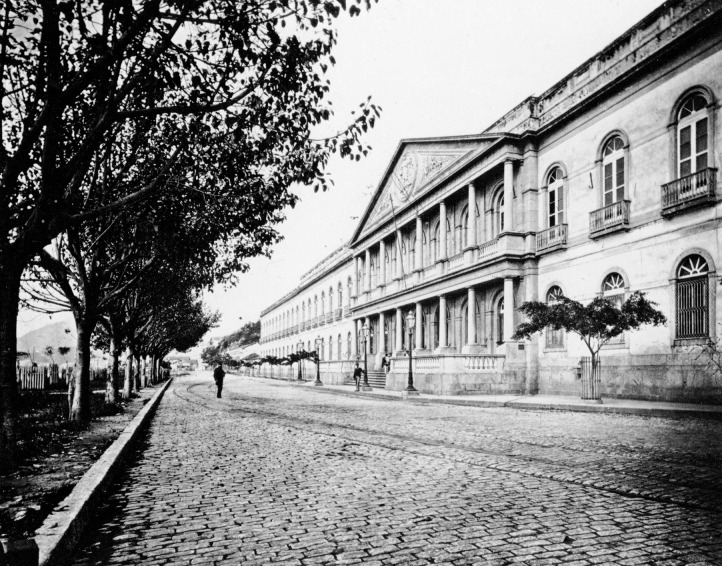
Faculty of Medicine of Rio Janeiro, which operated at the *Santa Casa de Misericórdia* building in Rio de Janeiro (Photograph Marc Ferrez), at Santa Luzia St, from its creation (December 5, 1808) until its transfer (October 12, 1918) to the beautiful building (also designed by the Portuguese architect Luis Moraes Júnior) at *Praia Vermelha* (surprisingly demolished in 1975) (photo from the *Instituto Moreira Sales* collection).

A quick summary of the beginning of Oswaldo Cruz’s professional trajectory should be the following: graduated in Medicine in 1892, the year his father died; replaced his father at the Corcovado textile factory; settled up a microscopy and clinical microbiology office in the downtown area of Rio, where he took care of his patients (Figure 4).

**FIGURE 4:**
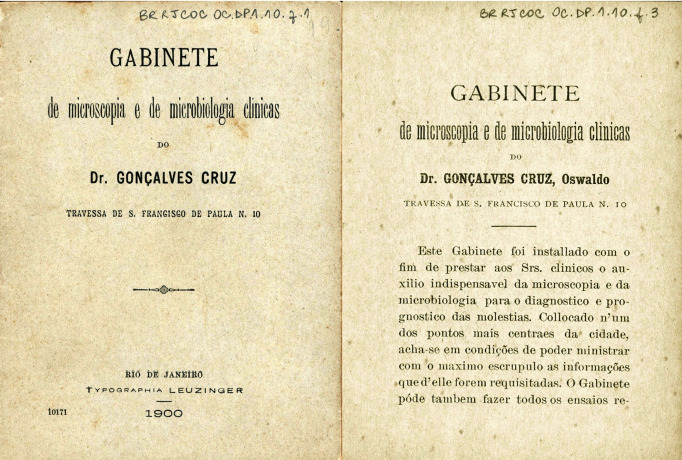
Leaflet of the Clinical Microscopy and Microbiology Oswaldo Cruz's office, located at *Travessa de São Francisco de Paula,* n^o^ 10 (document from the *Casa de Oswaldo Cruz* collection).

In 1893, one year after his graduation, Oswaldo married Emília Fonseca, aka Miloca (Figure 5), with whom he had five children. As a marriage gift, his father-in-law *Comendador* Fonseca, a wealthy Portuguese merchant, gave him a laboratory of clinical analysis, which was settled in his house’s basement, in the borough of *Jardim Botânico*. His children, as well as the dozens or maybe hundreds of his descendants displayed with pride “Oswaldo-Cruz” as their surname. Some say that “Liseta” (Elisa, 1893-1965) was his favourite… Bento (1895-1941, Figure 6) graduated at the Medicine Faculty, but he never worked as a physician - for some reason he became a banker; Hercília (1898-1968) was born when his father was in Paris for the course in the *Institut Pasteur*; Oswaldo Cruz Filho (1903-1977) graduated as a scientist and became president of *Fiocruz*, after its foundation (1970 to 1972); and Walter Oswaldo Cruz (1910-1967), who was a scientist too and a man of great value, was severely offended and persecuted by the military dictatorship^c^.

**FIGURE 5:**
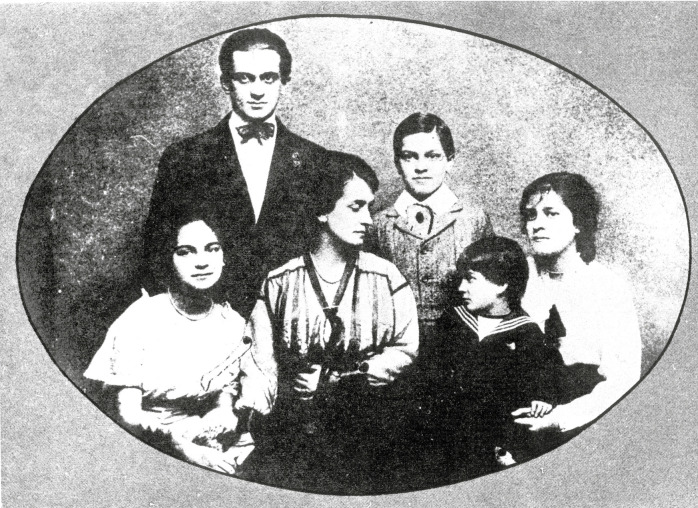
Emília Fonseca (Miloca), Oswaldo Cruz’s wife, and their five children: behind and standing, Bento Oswaldo Cruz and Oswaldo Cruz Filho, and, at the first row, Hercília, Miloca, Walter and Elisa “Liseta” Oswaldo Cruz (photo from the *Casa de Oswaldo Cruz* collection).

**FIGURE 6:**
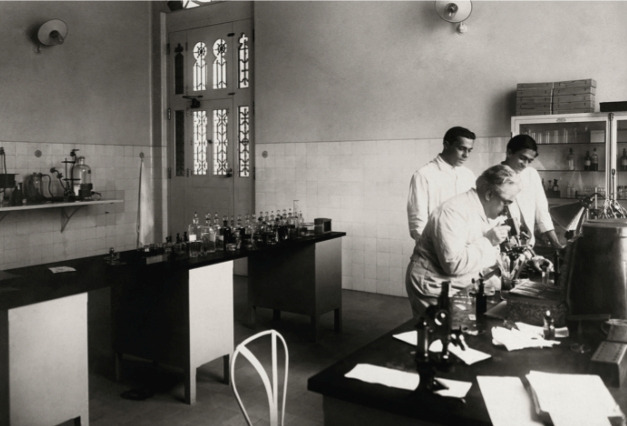
Oswaldo Gonçalves Cruz with his son Bento Oswaldo Cruz and assistant Burle de Figueiredo at Cruz’s laboratory on the second floor of the Moorish Castle (*Castelo Mourisco*). Although graduated as a physician, Bento never practiced medicine, and eventually became a banker. Surprisingly, he used to accompany Oswaldo Cruz to the lab quite often, and he shows beside his father on many pictures (photo from the *Casa de Oswaldo Cruz* collection).

Invited by Salles Guerra, Oswaldo Cruz built up and begun to coordinate the Clinical Analysis Laboratory of the *Policlínica Geral do Estado do Rio de Janeiro* ^d^, in 1894. During the same year, he was invited by Francisco Fajardo, who was then the director of the *Instituto Sanitário Federal* (Federal Sanitation Institute), to join a team formed also by Dr Eduardo Chapôt-Prévost, that should investigate a cholera outbreak in the region of *Vale do Paraíba*. As both Oswaldo Cruz and Chapôt-Prévost had laboratories at their homes, they were able to quickly detect the disease.

In 1897, through an appointment by Francisco de Castro (1857-1951), Oswaldo Cruz’s Propaedeutics teacher in the Medicine Faculty, he travelled to Paris with his wife and their two children, in order to attend the course of *Microbie Technique in the Institut Pasteur.* Oswaldo completed the course in the seventh year of its creation, which would be turned into the famous Course of *Microbiologie Générale.* Oswaldo Cruz benefited from a “fellowship” from the *Institut Pasteur* and had a good intellectual performance both in the course and in other activities he had simultaneously (intern at the Paris Toxicology Laboratory and at the Professor Félix Guyon's Urinary Tract Service, and trainee at a laboratory glassware factory), and established privileged relationships with other French scientists and physicians. Oswaldo Cruz never encountered Louis Pasteur (who had died two years before Oswaldo’s arrival), but, having stayed in Paris for about two years and three months, he was fortunate to meet and study with the first generation of pasteurians: Émile Roux, Émile Duclaux, Charles Chamberland. Elie Metchnikoff and Joseph Grancher - all of whom having made notable contributions to science and experimental medicine (Figure 7, Figure 8).

**FIGURE 7:**
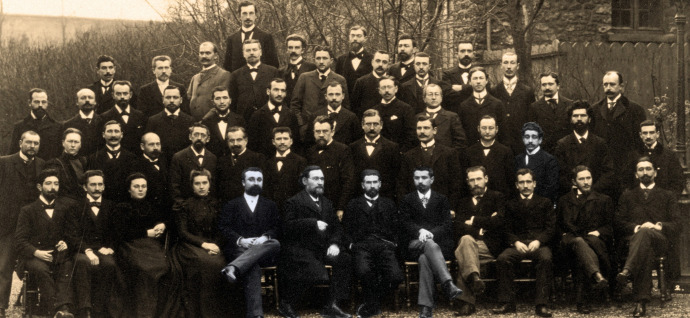
*Cours de Microbie Technique* at the *Institut Pasteur*, in Paris. Graduation of the 1898 class. From left to right, seated, Jean Binot (5th), Elie Metchnikoff (6th); Emile Roux (7th), Amédée Borrel (8th). In the row above, Oswaldo Cruz (3^rd^ from the right to the left). *Institut Pasteur* Collection. Oswaldo followed the course, which would later become the famous *Cours de Microbiologie Générale*, in the seventh year (1897-1898) of its creation (photo from the *Institut Pasteur* collection).

**FIGURE 8:**
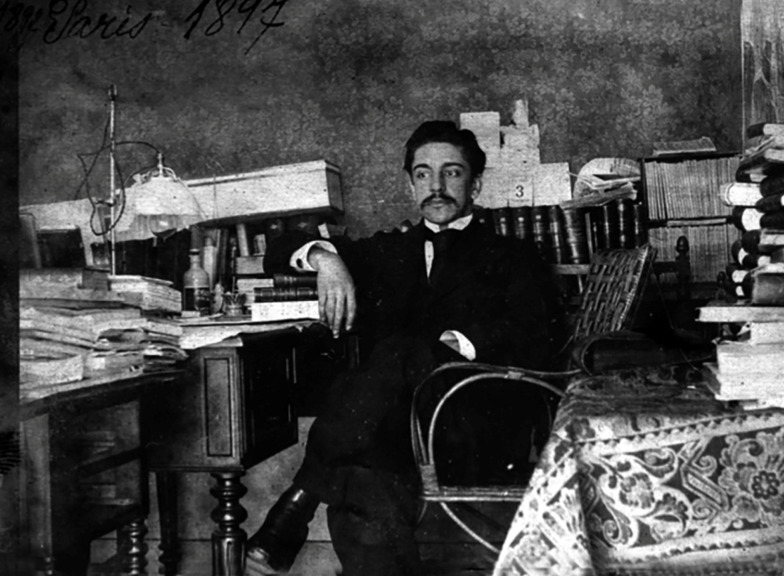
Oswaldo G Cruz in his office at the apartment at *Rue Marbeuf* in Paris in 1897 (photo from the *Casa de Oswaldo Cruz* collection).

Oswaldo Cruz became technical director of the *Instituto Soroterápico Federal* (1900-1902), created by the Baron of Pedro Afonso (1845-1920). After an argument between them, both decided to resign, but Nuno de Andrade, then in charge of the *Diretoria Geral de Saúde Pública*, accepted the Baron’s exoneration and eventually Oswaldo Cruz was named General Director of the Institute.

The scientist kept his position till 1916, when he moved to Petrópolis to become the mayor of that city. Oswaldo Cruz occupied simultaneously the positions of Director of the *Instituto de Manguinhos* and General Director of Public Health (1903-1909); when he left the latter, he pointed his former disciple Henrique de Figueiredo Vasconcelos (1875-1948) as his substitute. In 1907, Oswaldo Cruz won the golden medal at the XIV International Congress of Hygiene and Demography, hold in Berlin; when he returned, the city of Rio de Janeiro had gotten totally freed of yellow fever and plague. It was an interesting picture: in contrast with the hostility he had suffered by means of offences, harsh criticisms and newspaper charges, the population welcomed him enthusiastically when he returned from Europe, in 1908 (Figure 9). Awarded by the national medical corporation, Oswaldo Cruz took office in the *Academia Brasileira de de Letras* (Brazilian Literature Academy, *ABL*) in 1913, after refusing several invitations, as he considered himself a man “not suited to honorific titles nor homages”^e^. In 1914, when the First World War began, Oswaldo Cruz was in France. He had been named Officer of the Legion of France (*Legion d’Honneur*, Figure 10), a title that allowed him to move his family from Paris to England (Figure 11), where he considered they would be more protected. Oswaldo Cruz was so deeply revered in France that a 152-meter street close to Ranelagh subway station was named after him (Figure 12). It connects the Rue de Ranelagh to the Boulevard Beauséjour^8^.

**FIGURE 9:**
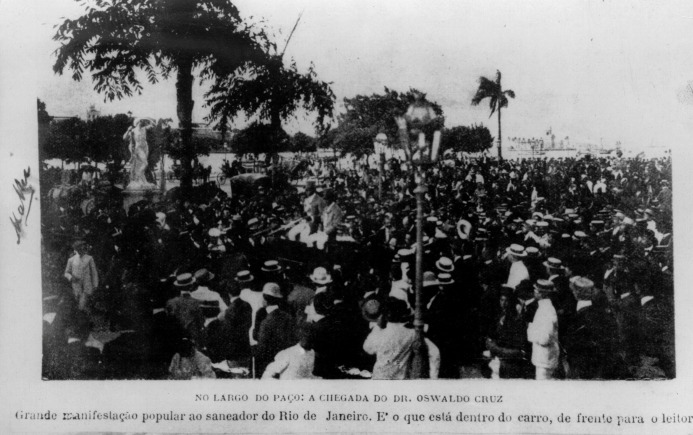
The very impressive image shows O Cruz being welcome and acclaimed, as a hero, when returning from Berlin (Germany, 1907) where he won the 1^st^ Prize of the Universal Exhibition on Health and Hygiene, which has had five million visitors, after have being vilified and satirized with cartoons, excessively hard, sometimes (photo from the *Casa de Oswaldo Cruz* collection).

**FIGURE 10:**
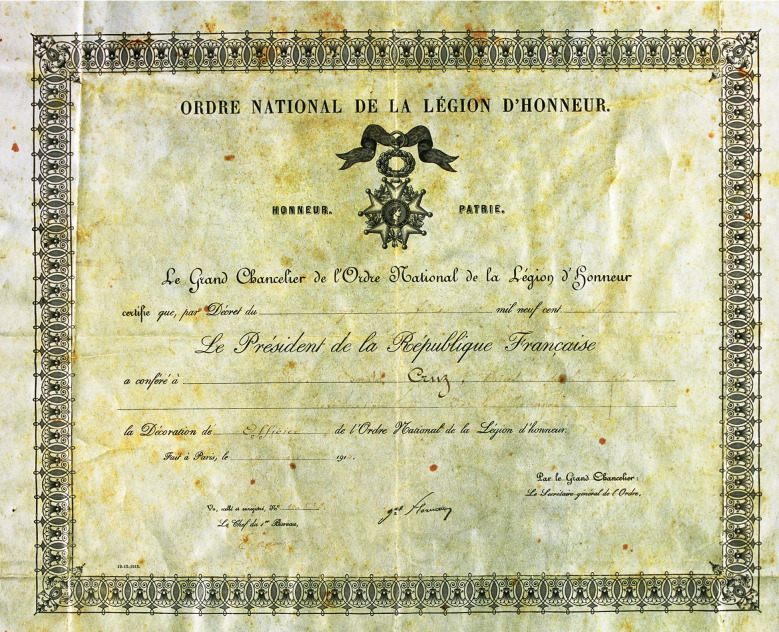
Diploma of *Officier de l’ordre de la Légion d’honneur* granted to Oswaldo Gonçalves Cruz, in Paris, by the President of the French Republic, *Grand Chancelier de l’ Ordre*, Mr. Raymond Poincaré, in 1914 (document from the *Casa de Oswaldo Cruz* collection).

**FIGURE 11:**
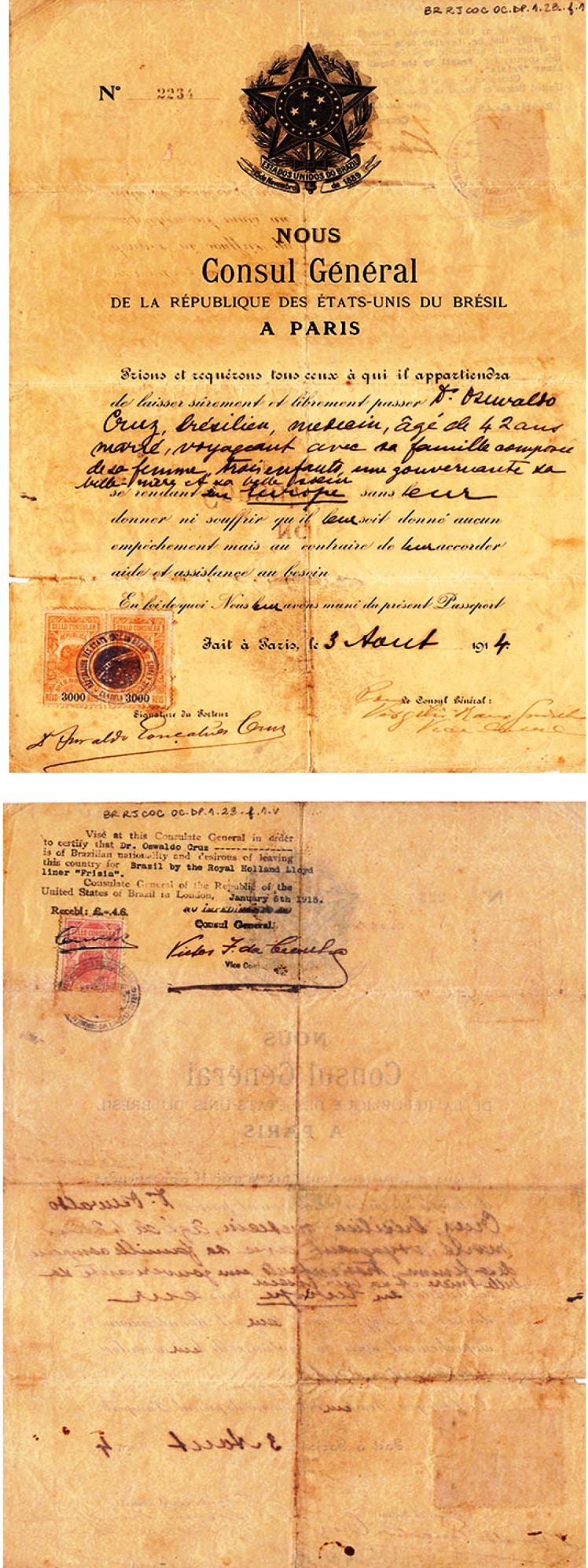
*Laisser-passer* given to Oswaldo Gonçalves Cruz, by the General Consul of the Federative Republic of Brazil in Paris on August 3, 1914. This document allowed Oswaldo Cruz moving, with his family, to London, where he estimated he would be safer (document from the *Casa de Oswaldo Cruz* collection).

**FIGURE 12:**
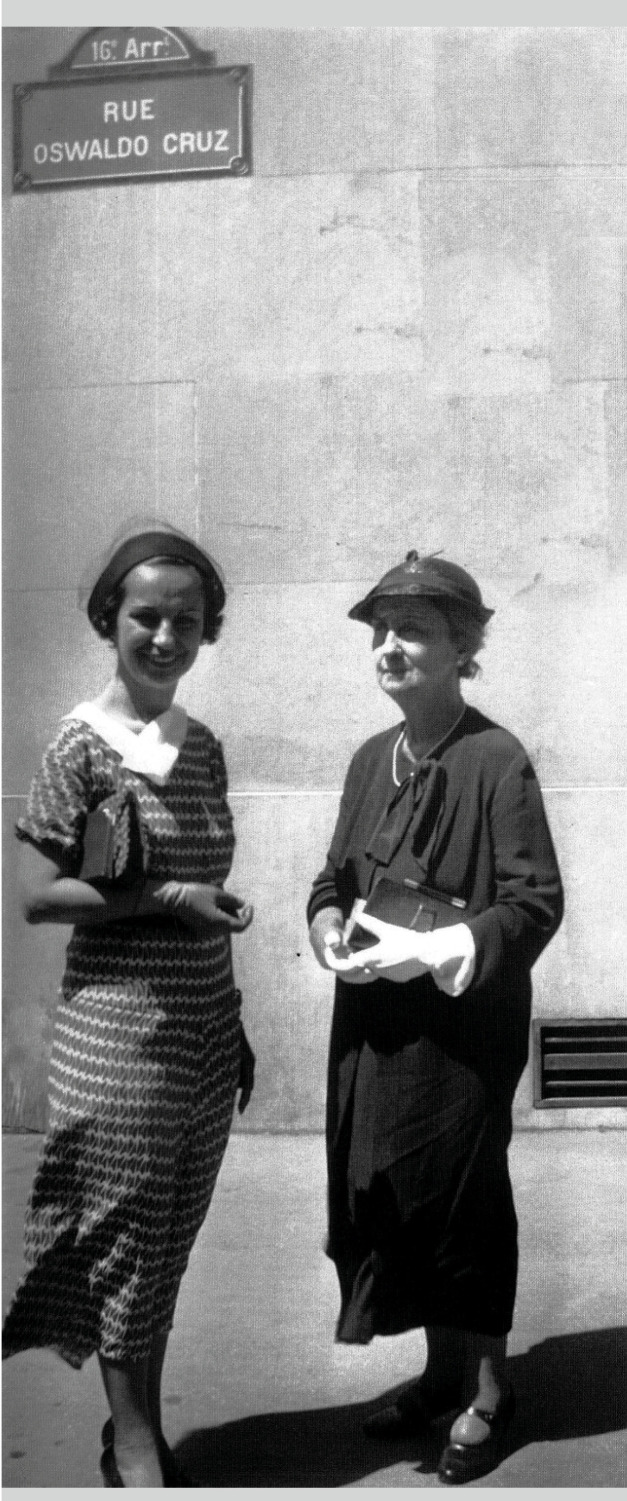
Miloca stands with her daughter, Liseta, below the sign of the Oswaldo Cruz St., a small street (152 meters long) near the Ranelagh Metro station, in Paris' *16th arrondissement*, connecting the *Rue de Ranelagh* to the *Boulevard Beauséjour* (photo from the *Casa de Oswaldo Cruz* collection).

A humble man, fully and permanently dedicated to study and work affairs, Oswaldo Cruz expressed the values and virtues he cultivated through the library hallmarks (*ex-libris*) he ordered (Figure 13) - one of them showing his name and the wording *“Saber, Querer, Poder, Esperar”* (to know, to want, to be able, to wait); the other displaying his famous saying *“Fé eterna na Ciência”* (Eternal faith in Science) in black and white letters, and his legendary motto*: “Não esmorecer para não desmerecer”* (Not to wane to not be belittled).

**FIGURE 13:**
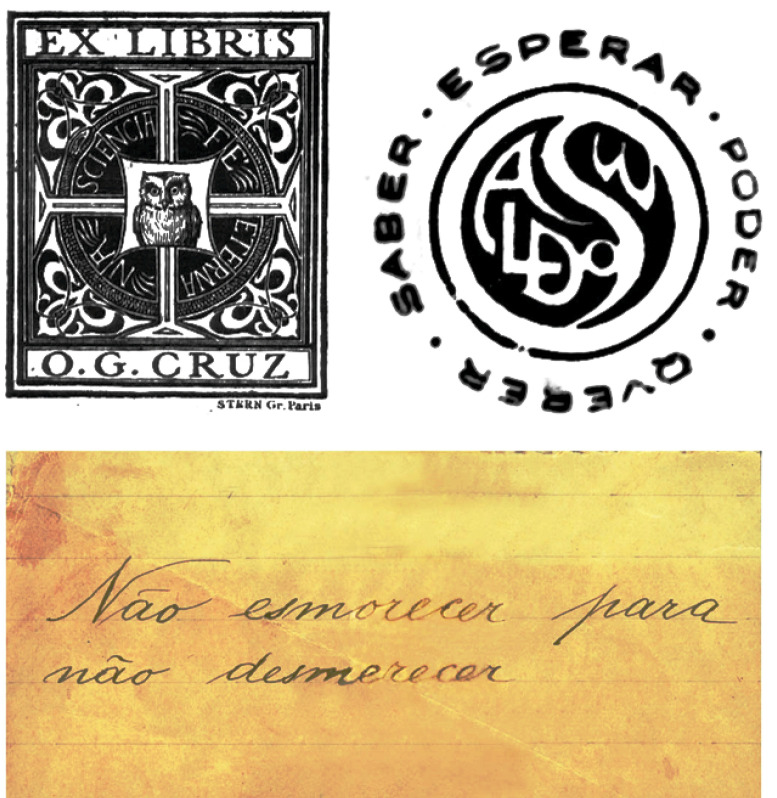
Two of the Oswaldo Cruz’s *Ex-libris*: “To know, to expect, to be able, to want” (*Saber, esperar, poder, querer*) and “Eternal faith in science” (*Fé eterna na ciência*) as well as the famous motto "Not to wane to not be belittled" (“*Não esmorecer para não desmerecer*”) (documents from the *Casa de Oswaldo Cruz* collection).

As a big fan of architecture and photography, Oswaldo Cruz got from his collaborators the nickname “*Jacinto*”, a character of the Portuguese author Eça de Queiroz (1845-1900)^9^ marked by his enthusiasm for technology. When he travelled to France^f^, sponsored by his father-in-law Fonseca - a famous, eminent, powerful businessman - Oswaldo acquired a stereo photography machine.

The following excerpt was extracted from a text by Carlos Chagas, written after Oswaldo Cruz’s death:

*The best features of Oswaldo Cruz’s moral individuality remain in the affective reminiscences of this House, firmly embedded in the feelings of the many of us who have experienced the benefits of his affection. He possessed the rare privilege of being simultaneously obeyed and respected, thus establishing the ascendency of his sovereign will by means of loyalty assurance and decisive personal affections. Every employee in Manguinhos, regardless his hierarchic position, could find in his master the best of friends and the steadiest support during setbacks and misfortunes. And by through work efforts and collective dedication they repaid the moral comfort of a tender, tolerant leadership, which ensured all their rights - and for that reason deserved the strict fulfilment of all duties…*

He describes what we can also observe through the many chapters of a book entitled *Oswaldo Cruz in the judgement of his contemporaries* ^10^, *which evoke Oswaldo’s human/humanistic attributes and virtues. Averse to publicity, he avoided the press; a restless research worker, he devoted his whole time to studies, work and family. He used to skip social life.*

One can be surely persuaded by such words that Oswaldo Cruz was an authoritative man, a real leader, but also respected and admired for his kindness and his respectful, friendly, warm, humanitarian approach to his subordinates. It seems that he was fond of children, and used to visit many schools… he showed himself specially interested in attending to the teachers’ classes. In fact, he was a humanist. Nevertheless, as he declared in his testament, he only became a religious man in the end of his life, probably under the influence of his wife Miloca, who was very religious^g^.

In 1907 many things happened in his life. He was still in charge as Public Health Director and, returning from a trip to Europe, he felt the first symptoms of nephritis, the same condition that had taken away his father’s life. Nevertheless, till his own death, Oswaldo Cruz would never avoid to take part in missions marked by hard and unhealthy conditions, as in the Amazon region, when he was called to tackle, in 1911 a malaria outbreak that flagellated the workers of the Madeira-Mamoré Railway. In short, it is not going too far to define him as a man who dedicated himself unreservedly to labour and to the love of neighbour.

One may wonder if scientists and scholars should be classified as genial or non-genial, or if they just need to be ranked by sheer luck… In fact, it is possible to consider that all good ideas are genial… although just a few of them would prove to be correct. Thus, it is possible that, besides a result of his exhaustive and dedicated work, the accuracy of his ideas and convictions may be due to chance. Oswaldo Cruz based his strategies to eliminate the yellow fever on the resolute conviction that Carlos Finlay’s proposal was correct. The Cuban researcher Finlay (1833-1915) believed that the transmission was made by mosquitoes^11^, while in our country, and in many others, the common belief was still that the diseases were the result of miasma emanations (the “bad air” in the etymology of *malaria*), instead of microbes. The whole control of the disease supported and executed by Oswaldo Cruz focused in fighting the vector and isolating the individuals that could contaminate it. There were also evidences gleaned by Emílio Ribas (1862-1925)^12^ in São Paulo showing the probable existence of a vector involved in the transmission of that illness. Apparently, the idea came up two and a half centuries before to the Portuguese physician João Ferreira da Rosa, when he was looking after the Marquis of Montebelo (1595-1662), who had caught the yellow fever, and begun to consider another way of transmission instead of through the air or the soil^13^.

René Laclette (1903-1979) was a standing Academician and Director of the Museum of the *ANM* during the first term of the President Academician José Leme Lopes (1904-1990), when Oswaldo Cruz Filho was the Director of *Fiocruz*. Laclete related that it was at *ANM* that the Academician Fernando Francisco da Costa Ferraz (1838-1907), then in his 54 years (and almost twice as old as Oswaldo Cruz, who was 30, and his great opponent), sustained that the yellow fever should be combated by keeping the soil clean. There were already many reasons to a hostile reaction against the prestige that Oswaldo Cruz acquired at the time from Francisco de Paula Rodrigues Alves. *ANM*’s manifest scepticism towards Oswaldo Cruz’s ideas was enough to foster a mood of national exacerbation and a large popular uproar, ending with the Military Academy Revolt in 1904. At the peak of the popular riot, when the population demonstrated in the streets by means of a widespread vandalism, which marked a grave and important moment in the nation’s history, Oswaldo Cruz handed in his resignation: “I do not feel able, within this popular dissatisfaction, to keep my measures.” The President then replied: “No, we do want the measures”. It could seem impressive the contrast between the tremendous popular dissatisfaction and the persevering reliance placed on Oswaldo Cruz by the authorities - President Rodrigues Alves, José Joaquim Seabra (1855-1942), then Interior Minister and afterwards Minister of Transport and Aviation in the following tenure, and Francisco Pereira Passos (1836-1913), the mayor who defined the whole urbanization of Rio based on a project drafted by the Baron of Mamoré (1825-1898). However, one must keep in mind that the main question at that time would rather be the international trade heavily affected by the then epidemic diseases that disturbed the port of Rio de Janeiro, and not exactly a presidential concern with the degree of popular satisfaction.

Obviously, Oswaldo Cruz had also good supporters - Sales Guerra, Chief of the Laboratory of Clinical Analysis in the *Policlínica Geral do Rio de Janeiro*, President Rodrigues Alves’s personal doctor and, later, Oswaldo Cruz’s biographer, was one of them… Émile Roux (1853-1933) himself, when asked by the Baron of Pedro Afonso (who had recently took over the direction of the *Instituto de Manguinhos*) about an indication of a competent technician for the position of General Director of Public Health, replied: “You have this man already; he is in Brazil now, and has graduated in our course. He is the right man to perform the work at the helm of the institute”. Massilon Saboia (1886-1974), one of Oswaldo Cruz’s collaborator and disciple, stated: “Cruz was assigned as General Director of Public Health by the invitation of the harsh Baron of Pedro Afonso and the gentleman Sales Guerra’s indication”^14^. Therefore, one can conclude that even as early as Saboia’s time, there were already completely different opinions concerning to Cruz’s personality - a man that accomplished in his short lifetime the following deeds: i) sanitation of the Rio de Janeiro city, and created the foundations and models to be applied to capitals like Belém; and ii) foundation of the *Instituto de Manguinhos*, which - as well as the *Institut Pasteur* - produced bioreagents, serums and vaccines, but was the template for experimental research and medicine. Carlos Chagas attributed to Oswaldo Cruz the creation of experimental medicine in Brazil^15^ but this is probably a too generous statement by the friend and admirer Chagas, since the bases for the development of experimental medicine in Brazil started to be established, even before Oswaldo Cruz, both in São Paulo and at the Faculty of Medicine of Rio de Janeiro, by figures such as Emílio Ribas, Adolpho Lutz and Vicente Cândido de Figueira Saboia, the Viscount of Saboia (*Visconde de Saboia*, 1835-1909).

Oswaldo Cruz eliminated the yellow fever and controlled the plague with the deployment of an army of sanitary agents (the so called *mata-mosquitos*, “mosquito killers”). He had estimated an ideal number of 1,200 men, but could enrol only 85… an unfortunate scenario that we could expect to find in the dawn of the last century but, unhappily, still persists nowadays, and, more surprisingly, is not restricted to developing countries... The rat extermination in the city counted on several strategies, including paying for dead rats to stimulate more people to hunt them - what made some swindlers begin to breed rats to sell them to the government. The compulsory vaccination decree (anti-smallpox), revoked in 1904, was one of the most unpopular laws. The measures to combat the yellow fever epidemics were adopted simultaneously and it was due to them that, during a visit to the USA in 1908, after the Exposition of Berlin - where he was awarded the golden medal after competing with 123 countries - Oswaldo Cruz declared to Theodore Roosevelt (1858-1919) that the American fleet could berth safely again in the Brazilian ports, because the yellow fever was eradicated from the city of Rio de Janeiro.

Elected in June, Oswaldo Cruz was appointed as Full Member of *ANM* in August, 24^th^,1899^h^, becoming the Patron of the institution’s 90^th^ Chair^i^. The Sections of *ANM* were created in the Statute of 1835, when the Society of Medicine and Surgery became to be called Imperial Academy of Medicine. Oswaldo Cruz entered *ANM* probably in the Section of Public Health [a section that existed since 1898. In 1906 he was transferred to the Section of Sciences Applied to Medicine and Pharmacology (created in 1902), which would be presided by him for three mandates, from 1913 to 1916^j^. João Baptista de Lacerda (1846-1915)^k^ announced the admission of Oswaldo Cruz at the *ANM* in the *Brazil Médico*, a contemporary scientific journal, listing his (then few) academic publications. Clearly, at the beginning of the XX century, the actions and motivations that guided both scientific practice and the individual profiles of researchers, who were still scarce in Brazil, were very different from those that are in force today. Notwithstanding, it could be opportune to remember that, in addition to being not only a well-formed and creative scientist, but a researcher committed with the study of public health problems, Oswaldo Cruz was also an administrator, leading scientifically the *Instituto Oswaldo Cruz* and the *Diretoria Geral de Saúde Pública* at a standard of excellence. These aspects of Oswaldo Cruz activities, that could compete with and even hamper his scientific and teaching tasks, would probably be overlooked nowadays by strict “research productivity” criteria of the National scientific funding agencies.

A nice story to be told is the origin of the *Castelo Mourisco* (“Moorish Castle”), the present headquarters of the *Fundação Oswaldo Cruz*, which was conceived through this original drawing by Oswaldo Cruz himself. Infatuated with architecture and photography, Oswaldo had a book of 1907 about Grenada and the palace of Alhambra (Figure 14a), whose details had previously inspired him (Renato Gama Costa, Architect Researcher, *Casa de Oswaldo Cruz*, personal communication). He drafted (Figure 14b) a building (resembling a sketch of a medieval palace) and showed the drawing to the Portuguese architect Luiz Moraes Júnior (1868-1955)^l^, who took up the mission of translating Oswaldo Cruz’s dreams, desires and insights into a feasible project. Apparently, when travelling to the Berlin Exposition in 1907 they visited the Berlin Synagogue (Figure 14c,1866), whose towers closely resemble our Moorish castle of the *Fundação Oswaldo Cruz*. The building showed in the following picture is the New York Synagogue (Figure 14d, 1872), a construction inspired on the Berlin Synagogue (Neue Synagoge). Thus, both of them (specially Berlin’s), and the *Observatoire de Montsouris* or *Palais du Bardo* (Figure 14e, 1867), located at the *Parc de Montsouris* in Paris, along with the Palace of Alhambra have inspired Moraes Júnior and Oswaldo Cruz to create the magnificent three-storey Moorish Castle building (Figure 14f, 1918), a place worth visiting, according to the authors, now two vintage employees. This story has been told in different places, including more recently by two former directors of the *Instituto Oswaldo Cruz (IOC)* ^16^.

**FIGURE 14:**
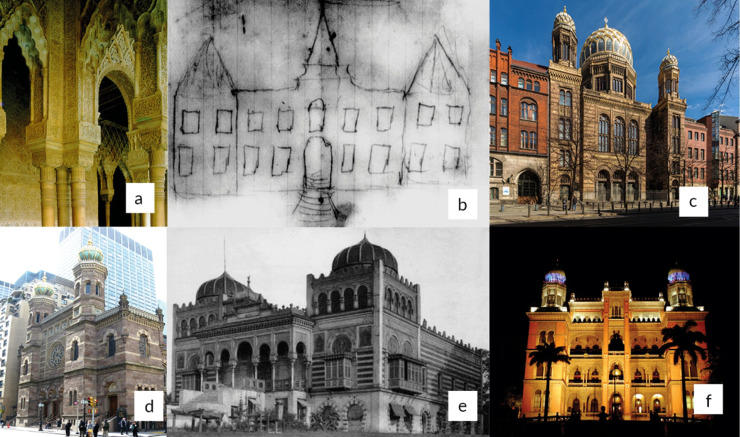
(a) Detail of the Alhambra Palace, Granada, Spain (photo: Renato Gama da Rosa) (b) Draft made by Oswaldo Gonçalves Cruz, himself, to transmit to the Portuguese Architect Luiz Moraes Júnior the idea he had in mind to the main building of the *Instituto de Manguinhos* in the (c) The Synagogue of Berlin (design: Eduard Knoblauch, 1865; photo: Ansgar Koreng), available at https://commons.wikimedia.org/wiki/File:Neue_Synagoge,_Berlin-Mitte,_160328,_ako.jpg. File licensed under the Creative Commons Attribution 3.0 Germany license; (d) The Synagogue of New York (design: Henri Fernbach, 1872; Photo: Jim Hendersonor), available at https://commons.wikimedia.org/wiki/File:Central_Synagogue_Lex_jeh.jpg under the Dedication to the Universal Public Domain Creative Commons CC0 1.0. (e) The Montsouris Observatory - *L’Observatoire du parc Montsouris, Le Palais du Bardo,* Paris 14e - (design : Otapon to represent Tunisia in the *Exposition Universelle*, 1867), reproduced part of the “*Palais du Bey de Tunis*” that was reacquired from the Diwan of the Turkish militia in 1643. A fire completely destroyed it in 1998. Available from http://paris1900.lartnouveau.com/paris14/parc_montsouris/palais_du_bardo.htm, accessed on April 12^th^2020, and (f) The Moorish Castle - Castelo Mourisco - (design: Luiz Moraes Júnior, 1918) of the *Instituto Oswaldo Cruz*, present headquarter of *the Fundação Oswaldo Cruz* (Fiocruz) (photo André AZ & Peter Ilicciev, Coordenadoria de Comunicação Social, Fiocruz).

Wladimir Lobato Paraense (1914-2012)^m^ ^,^ ^17^, an admirable character and outstanding scientist of *IOC*, has narrated a story that deserves to be registered here. Lobato told that he was once accompanying Evandro Chagas (1905-1940) to the office of Director Antonio Cardoso Fontes (1879-1943), when he listened to an unusual conversation. At the time, there was a flying club (Figure 15) in the vicinity of the campus - in fact, not far from the Castle, and an Airforce Colonel had just requested the *IOC* director to “please, tear down the Castle towers, because they threaten our airplane landings”. Suddenly, Evandro Chagas came up with a prompt reply: “Colonel, without the towers your pilots will never find the way to the track.” And that was the end of the Colonel’s claims.

**FIGURE 15:**
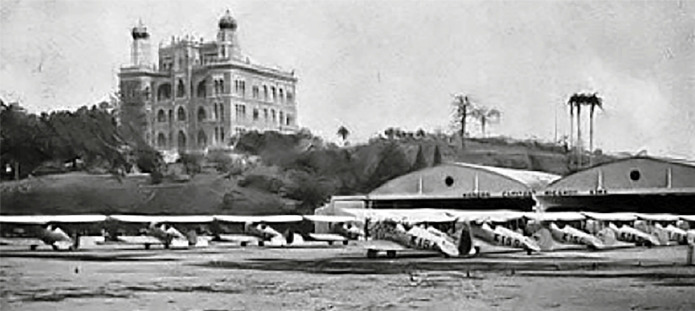
The flying club existing in the vicinity of the Manguinhos campus, in fact, not far from the Castle. See the text for an anecdotical story about it.

In a speech during the celebration of the 95^th^ anniversary of the *IOC*, one of us (CTDR, then the Director)^16^ commented the daily work routine in *IOC* before the inauguration of the Castle^n^ (though other buildings had been finished before). The researchers used to arrive in horse-drawn carts (Figure 16) or on horseback, which they picked up on the (now called) Leopoldo Bulhões street, after taking a train at *Praça da República* railway station.

**FIGURE 16:**
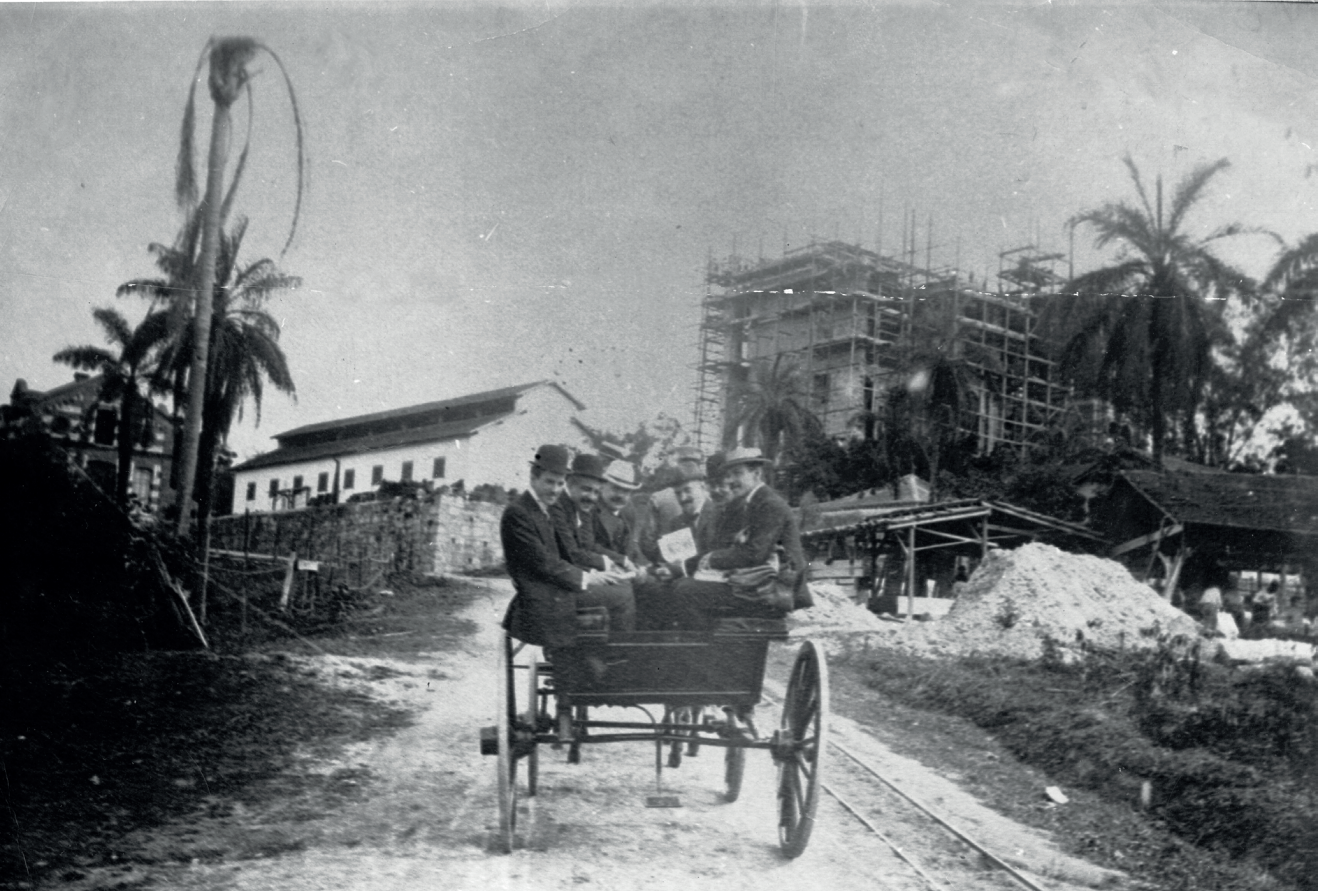
Researchers of the Instituto Oswaldo Cruz arriving in Manguinhos by a buggy. From left to right: Oswaldo Cruz (2^nd^), Gustav Giemsa (3^rd^), Stanislas von Prowazek (4^th^) (photo from the *Casa de Oswaldo Cruz* collection).

*“This morning, while the dawn colours were still vague, I found myself thinking on what would be happening in this same place precisely 95 years ago, when by order of President Campos Salles, the* Instituto Soroterápico Municipal *(which soon would be Federal) was founded. I imagined a few horse-drawn carts bringing the Baron of Pedro Afonso, some assistants, movement, packages, papers, test tubes, some students… It should be terrible to step in the swampy ground with boots. Oswaldo Cruz, the technical director of the institute, Ezequiel Dias, the Baron’s aide at the* Instituto Vacínico*, Ismael da Rocha (1858-1924), transferred from the Army health service, Figueiredo de Vasconcellos, the Baron’s assistant physician… I wonder how many assistants those geniuses needed… Lobato Paraense told me that the facilities - assuming it could be called so - of transport were not available since the first times. It seems that in the beginning of our history the main access to the campus was by train. The researchers met at* Praça da República *to take the 10:30 AM train. They arrived at São Francisco Xavier railway station in 20 minutes. From there, they took another train in the line that is currently called Leopoldina, and within 10 minutes they would reach the gatehouse of Leopoldo Bulhões street. At the entrance, three horses were waiting for the most graduated scientists. The maritime route was another option, by a vessel belonging to the* Repartição Fiscalizadora da Pesca *(Fishery Inspection Department) which was docked just behind the Health Ministry building, on Brazil Avenue. Through those crooked ways the researcher arrived at two rugged houses in the campus - one of them on the hill between the Evandro Chagas Hospital and the Rocha Lima Pavilion, where the plague vaccine was produced. The other house was larger and near the current Moorish buildings. The researchers used to eat at the veranda. At noon they stopped for lunch. The table was a door supported by two empty barrels and was partially covered by a coarse tablecloth, with two wooden benches on each side. Everybody had to rush, for food was not abundant: a classic potato and chicken stew, rice, bread and, finally, some bananas and weak coffee. The latecomers would only find bones and some grains of rice. There was no supper, and unless you had brought some takeout from home, you would have to make do with the plenty fruit that could be reaped throughout the campus”.*

There are some lists with the names of the first collaborators. According to Carlos Chagas, the hard core that begun with Oswaldo Cruz included Antonio Cardoso Fontes, Ezequiel Dias (Oswaldo’s brother-in-law), Henrique Figueiredo de Vasconcelos (who succeeded Oswaldo Cruz as Director of Public Health), Adolpho Lutz (1855-1940), Gaspar Vianna (1885-1914), Gustav Giemsa (1867-1948), Herman Duerck (1869-1941), Max Hartmann (1876-1972) and Stanislas Von Prowazek (1875-1915). According to René Laclette the list must also comprise Arthur Neiva, Carlos Chagas, José Gomes de Faria (1887-1962), Henrique Aragão and Rocha Lima (1859-1956). Massillon Saboia enumerated the staff with sufficient training to deal with microscopy and microbiology: Adolpho Lutz (1855-1940), Emílio Ribas (1862-1925), Martin Ficker (1868-1950), Luiz Pereira Barreto (1840-1923), Pedro Severiano de Magalhães (1850-1927) and Alfredo Carneiro Ribeiro da Luz (1852-1931). Representative pictograms of the hardcore of Oswald Cruz’s team at different moments are shown aside and on the next page (Figure 17, Figure 18).

**FIGURE 17:**
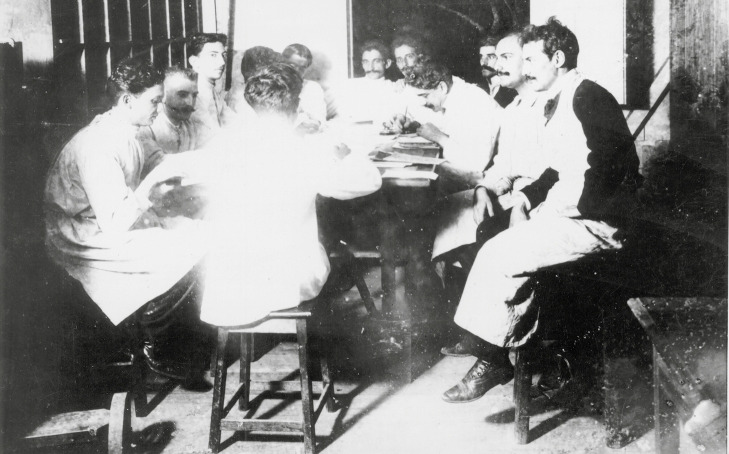
The photography of Oswaldo Cruz and team was taken in 1904, in the shed used as library and photograph room. The scientists used to attend the space on Wednesdays, for the scientific meeting of the *Instituto Soroterápico*. From behind: Alcides Godoy (1880-1950) and, on his right, anti-clockwise, Antônio Cardoso Fontes, Henrique da Rocha Lima, Oswaldo Cruz, Henrique Marques Lisboa (1876-1967), Carlos Chagas, Ezequiel Caetano Dias, Rodolpho de Abreu Filho, Paulo Parreiras Horta (1884-1961), Henrique Aragão e Afonso MacDowell (1881-1958) (photo from the *Casa de Oswaldo Cruz* collection), http://arch.coc.fiocruz.br/index.php/oswaldo-cruz-reunido.

**FIGURE 18:**
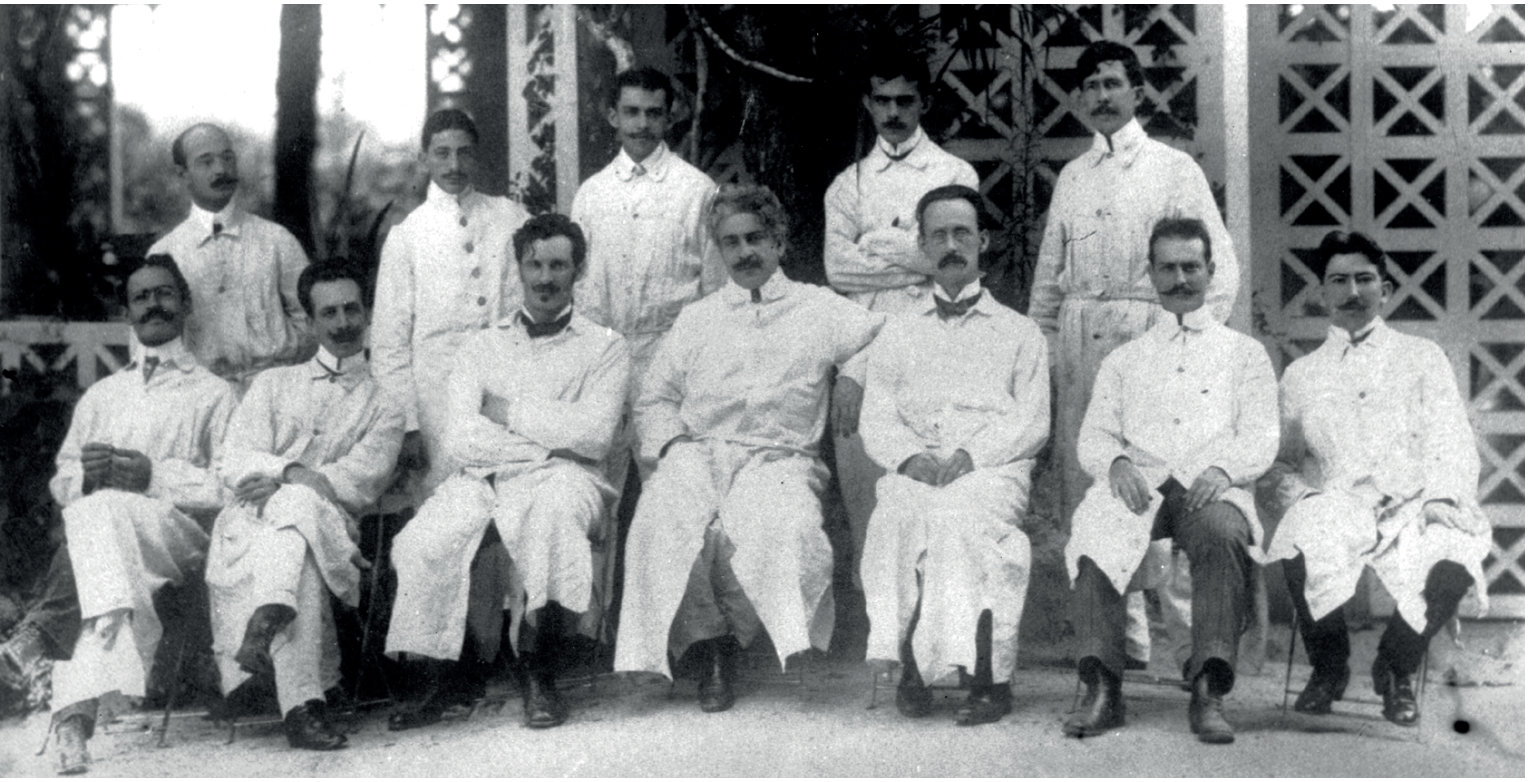
First generation of scientists from *Manguinhos*, in front of the Tea House, where we may still have lunch at Fiocruz today (when the place is not under renovations). From left to right, standing: Arthur Neiva, (not identified), Gaspar Vianna, Astrogildo Machado and Alcides Godoy. Seated: José Gomes de Farias, Carlos Chagas, (not identified), Oswaldo Cruz, Adolpho Lutz, Cardoso Fontes and Parreiras Horta (photo from the *Casa de Oswaldo Cruz* collection).

Other major contribution by Oswaldo Cruz was the malaria control in the Madeira-Mamoré railway region. It is estimated that when Oswaldo Cruz arrived to coordinate the sanitation of the region, there was one death due to malaria to every railway sleeper of the track bed. He drastically reduced the casualties, even though the total number has been estimated at around 33,000, we don’t know for sure if it is a realistic number). It is interesting to note that the construction of a railroad in the territory of Rondônia in the beginning of the XX Century (circa 1900) was considered impossible. Someone had even declared that, even if all the world’s money and half the global population were involved in the work, such an endeavour would not be feasible. That’s when an American entrepreneur called Percival Farquhar (1864-1953) decided to accept the challenge, declaring: “It will be my business card”. And he indeed sponsored the work, whose real importance is hard to avail. The Madeira-Mamoré railway was part of a deal made with Bolivia by the chancellor Baron of Rio Branco. By its terms, that country should grant Brazil the territory of Acre, already occupied by Brazilians. In return, Brazil should offer an exit to sea, in order to ship Bolivia’s rubber production. The solution would be navigating through the Amazon River, but to get there it was necessary to access the Madeira River, which was navigable only over part of its course. So, the railroad would be the way of access to the river.

It's likely that René Laclette has correctly understood^o^ that the more suffering part of Oswaldo Cruz’s life may have been in health education: “...the Yellow Fever campaign in Rio de Janeiro, in which the struggle was more arduous against misunderstanding than against the disease”. Oswaldo would appear to be strong, courageous and obstinate in the pursuit of his ideals. It makes sense that one of his mottoes was “Not to wane to not be belittled”.

Although Oswaldo Cruz's best-known accomplishment, while at the helm of the Directorate General of Public Health between 1903 and 1909, is the fight against epidemics in Rio de Janeiro, he did also turn attention to the rest of the country. In the beginning of 1905, Oswaldo Cruz alerted the Minister J.J. Seabra to the need to protect Brazilian ports against the invasion of diseases such as plague and cholera. Brazil was a signatory to an agreement signed at the Paris International Convention in 1903, which obliged all member countries to strictly monitor their ports in order to protect themselves from those diseases. Thus, Oswaldo Cruz launched an expedition to the main sea and river ports in Brazil, which would take place in two stages: first, Oswaldo Cruz should visit 26 ports in the Southeast, North and Northeast^p^; the second stage started in the following year and was destined for southern ports^q^. In addition to the reformulation of port health services, Oswaldo Cruz also intended to build isolation hospitals and disinfection stations in the places visited. In this sense, the expedition was a disappointment, due to lack of support from the Ministry of Justice and Interior Affairs^18^ ^,^ ^19^. However, from this expedition it was possible to start drawing a map of health conditions in Brazil, from the hinterlands to the coast. In the following years, researchers from Manguinhos, such as Carlos Chagas, Belisário Penna, Adolpho Lutz, Arthur Neiva, in addition to Oswaldo Cruz himself, sought this unknown Brazil through the Amazon Basin, the São Francisco River Valley, the meeting of the Madeira and Mamoré rivers, among other remote parts of the country. These study trips provided subsidies for future national health policies, developed from the National Department of Public Health, created in 1920, and later absorbed by the Ministry of Education and Health, created in 1930. We can thus say that these actions corresponded to one of the most important legacies left by Oswaldo Cruz and by the first generation of scientists who have acted since the beginnings of the *Instituto de Manguinhos* era.

The UNESCO recently approved the creation of the "Oswaldo Cruz Chair of Science, Health and Culture"^20^proposed in 2019 by Fiocruz, through *Casa de Oswaldo Cruz*, a Fiocruz’s Technical-Scientific Unit dedicated to research and teaching in history of health and science and the preservation of the cultural heritage. The approval of the UNESCO Chair expands the possibilities for international exchanges and partnerships of the unit’s postgraduate programs with other Teaching and Research Institutions on topics related to their areas of expertise. It also represents the recognition of the need to articulate science and culture in addressing the country's health issues, once more inspired on the example of Oswaldo Cruz.

One should finally note that Oswaldo Cruz’s performance in the fight against the yellow fever pandemic represented, at that moment, a great innovation in relation to the methods of combating this disease and facing an epidemic. The idea of transmission through a vector had been tested to stop the Cuban epidemic and showed successful for the first time. Thereafter, Oswaldo Cruz, in Rio de Janeiro, and Emílio Ribas, in São Paulo, began studies to prove the effectiveness of what was known at the time as the “Havana theory”. Based on scientific evidence, Oswaldo Cruz, in charge of the *Diretoria Geral de Saúde Pública*, created the Yellow Fever Prophylaxis Service by implementing a series of sanitary measures aimed at fighting the mosquito, identifying cases and isolating patients either at home or at the São Sebastião Hospital, with screens and protections against mosquitoes. The scientist also launched a campaign by the newspapers to inform the population about ways of prevention. It was essential, then, to have the support of the federal government, especially when scientific evidence pointed to the need to adopt unpopular measures. More than 100 years later, and again facing a new devastating epidemic (Covid-19), it is almost impossible not to think about the huge setback we are currently experiencing…

## NOTES

a This article results from a speech given by one of us (CTDR, on “*Oswaldo Gonçalves Cruz: o personagem, o cientista e o Acadêmico*”, available from 46’08” to 1h17’30” at https://www.youtube.com/watch?v=I1B9eX9-hGU&feature=youtu.be, accessed on Apr14^th^, 2020) at the Brazilian *Academia Nacional de Medicina* at its Scientific Session held on August 3, 2017. The session had as theme “Oswaldo Cruz: Researcher and Sanitarist”, paying homage to the Academician Oswaldo Gonçalves Cruz in the centenary of his death (February 11, 1917) and because of the proximity of his birthday date (August 5, 1872), when we celebrate, as well, the National Health Day in our country.

b Bento was considered a tender, resolute, exemplary person. One day, when he found out that Oswaldo was smoking, he brought it up in a conversation mentioning that it wasn’t a good thing, not healthy, et cetera… Oswaldo listened to him in his very peculiar way... silent, quiet, an obedient look… till a day that his father caught him smoking again, and scolded him. And Oswaldo, as the young rebel that each of us had inside at the proper age, replies: “But *you* do smoke…” Bento, then, quit the habit to be able to request his son not to adhere to it. Bento possessed a severe disciplinary instinct. Once, Oswaldo was called to come back home from school in a hurry; when he arrived, his father ordered him to make his bed before leaving, because he had left it undone. Oswaldo referred to his father as “counsellor”. Paulo Gadelha describes him as a great friend of Oswaldo’s. He died hours before Oswaldo’s graduation; it was common to find Oswaldo at the São João Batista cemetery praying and talking, as if taking advice from his father.

c Some accounts by Emmanuel Dias are really impressive… as when Walter, after arriving to his laboratory, had the energy and gas supply shut off by saboteurs… to hinder his work… Walter didn’t use to take vacations regularly, and after a long period of non-stop working routine, he died on his first day of vacation.

d Currently presided by the Academician Omar Lupi da Rosa Santos (1967-).

e At some point, an *ABL* Academician had declared that Oswaldo was apparently putting his own glory above the Academy, what wouldn’t be reasonable. Oswaldo decided then to accept the insistent invitation and became full member of the *Academia Brasileira de Letras*.

f Oswaldo Cruz spoke French, English and Spanish, having studied German, as well. He took part of the group “*Germanófilos*”, formed by Dr Egydio Sales Guerra, Silva Araújo, Werneck Machado and Alfredo Porto.

g In his will, Oswaldo Cruz declared: “I die as a Christian, a passionate admirer of the Immortal Nazarene’s doctrines”. Testament of Oswaldo Cruz. Oswaldo Cruz’s personal archive. *Casa de Oswaldo Cruz* Collection.

h The Academician Antônio José Pereira da Silva Araújo (1853-1900) was then President of the *ANM*.

i The ANM 90^th^ Chair is presently held by the Academician Gilberto de Nucci (1958-).

j After O. Cruz’s death in 1917, the position of Full Academician at *ANM* was offered in August and occupied in October by the Academician Gustavo Koehler Riedel (1887-1934), who stayed in charge till his death, on May of 1934. Since 1979, the Statute confers on ANM three sections: Medicine, Surgery and Sciences Applied to Medicine.

k João Baptista de Lacerda is the Patron of Chair 87 (presently held by CTDR) and was former president of *ANM* from 1893 to 1895.

l At the time, L. Moraes Júnior was working in a restoration of Penha Church, and he and Oswaldo used to take the same train every day. There, they met for the first time. Benchimol, J.L. Manguinhos do sonho à vida. A Belle Époque da ciência. Rio de Janeiro, Casa do Oswaldo Cruz. 1990. 248 p.

m At IOC, we all had an immense admiration and affection for Lobato, a humorous guy that, although didn’t use to laugh often, would easily make his wife laugh with the jokes he cracked at the IOC Advisory Board meetings. Nevertheless, CTDR watched him shedding a tear just once: when he received in 2000, through CTDR’s and Hooman Momen’s (1952-) hands, the Henrique Aragão Medal, a commendation they created in 1991. Lobato said at the time: “It is curious to be awarded a medal named after the person who prevented me from undertaking the work of my life.” Lobato considered that the plasmodium’s biological cycle should include a hepatic phase [exo (pre) erithrocitic], as Henrique de Beaurepaire Rohan Aragão (1879-1956) had demonstrated in relation to the pigeon’s protozoan corresponding to plasmodium, *Haemoproteus columbae.* Aragão admonished Lobato: “Hey, stop it, halt this work, it’s a foolish thing.” Arthur Neiva (1880-1943), the first researcher in the world to describe the plasmodium’s chemoresistance, said: “Read Schaudinn’s work (the great German zoologist Fritz Richard Shaudinn, whose name was given to a prize that would be awarded to Carlos Justiniano Ribeiro Chagas; he has already demonstrated that it’s not like that by a series of photomicrographs.” Schaudinn supposed that he had seen (and in fact he documented, by drawings, not photos), a sporozoite (the infecting form of plasmodium injected by the mosquito bite) penetrating the red cell, the protozoan’s host cell in blood (what we currently know that is not possible). Therefore, Aragão insisted: “Lobato, it’s already proven that the sporozoites penetrate the red blood cells”, but Lobato replied: “No. They’re just drawings, I don’t believe them. Come to see my ideas.” But Aragão thought that Lobato was insisting on a silliness; he didn’t support his works, nor accepted to take a look at his results, while Lobato refused to stop his surveys. Then one day Aragão decided to visit Lobato at his lab. When he was about to leave the room, he asked: “Are the slides here?” He turned back, examined the slides on the microscope and declared: “It looks good, you can go ahead. I think you’re right.” But, soon after, the British scientist Percy Cyril Claude Garnham (Garnham, PCC 1966. *Malaria Parasites and Other Haemosporidia*, Blackwell Scientific Publications, Oxford, 1114 pp) would describe the hepatic forms, subtracting from Lobato Paraense the primacy of the breakthrough (maybe his life’s one). Nevertheless, Lobato achieved very important contributions for a general comprehension of a plasmodium exoerythrocytic cycle in mammals. This story was told in detail by Lobato Paraense himself during his speech after receiving the Henrique Medal, and was published in the *Memórias do Instituto Oswaldo Cruz* ^17^.

n The Castle begun to be built in 1905 and was finished in 1918, though there are photos showing Oswaldo Cruz working at his lab in the building as far back as 1910.

o Opening Speech at the Oswaldo Cruz Exhibition, during the 12th National Congress on Medicine, on August 24, 1972, the centenary of Oswaldo Cruz’s birth, available at the Archives of the *Academia Nacional de Medicina*.

p Cabo Frio in Rio de Janeiro; Vitória in Espírito Santo; Caravelas, Santa Cruz, Porto Seguro and Salvador in Bahia; Aracaju in Sergipe; Penedo and Maceió in Alagoas; Tamandaré and Recife in Pernambuco; Cabedelo and Paraíba in Paraíba; Natal, Mossoró, Areia Branca and Macau in Rio Grande do Norte; Camocim and Fortaleza in Ceará; Amarração in Piauí; São Luís in Maranhão; Belém, Óbidos and Santarém in Pará; Parintins and Manaus in Amazonas.

q Paranaguá in Paraná; São Francisco do Sul, Itajaí and Florianópolis in Santa Catarina; and Rio Grande in Rio Grande do Sul. He extended the trip to the capitals of Uruguay, Argentina and Paraguay and then to Corumbá, in Mato Grosso.
